# Childhood Adversity and Risky Behaviors among Chinese Rural Young Adults: The Mediation Effect of Perceived Stress and Moderation Effect of Social Support

**DOI:** 10.3390/ijerph192013194

**Published:** 2022-10-13

**Authors:** Lixia Zhang, Liwei Zhang, Alexander Testa

**Affiliations:** 1 Kent School of Social Work and Family Science, University of Louisville, Louisville, KY 40292, USA; 2 Brown School, Washington University in St. Louis, St. Louis, MO 63130, USA; 3 Department of Management, Policy and Community Health, University of Texas Health Science Center at Houston, Houston, TX 77030, USA

**Keywords:** adverse childhood experiences, risky behaviors, perceived stress, social support

## Abstract

Research has documented that adverse childhood experiences (ACEs) significantly impact young people’s risky behaviors. Yet, few studies have explored if individuals’ perceived stress mediates the association between ACEs and risky behaviors; also if social support moderates the pathways from ACEs to risky behaviors through perceived stress. This study aimed to assess the mediation effect of perceived stress and the moderation effect of social support in the study of ACEs and risky behaviors. This study sample includes 1091 Chinese rural young people. A web-based survey was used to assess demographic information, ACEs, perceived stress, social support, and seven types of risky behaviors. Multivariate regression models were run to test associations between ACEs and different risky behaviors while controlling for confounding variables. The mediation model (Model 4) and the moderated mediation model (Model 58) were also performed using the PROCESS macro. Multivariate regression results showed that, with increasing ACEs values, there was an increased likelihood of all risky behaviors. The moderated mediation analysis confirmed that perceived stress mediated the linkage between ACEs and risky behaviors. However, no significant moderating effect of social support was found. The study findings indicate that ACEs, risky behaviors, and perceived stress are significant public health problems among rural Chinese young people. Culturally appropriate prevention and intervention programs and services need to be implemented to address these issues and promote rural Chinese young people’s wellbeing and development.

## 1. Introduction

Risky behaviors refer to activities that harm one’s health and wellbeing and contribute to the risk of injury and disease. Such behaviors commonly include violence, early onset or unsafe sexual behaviors, substance use, unhealthy dietary behaviors, and inadequate physical activity (e.g., screen-based sedentary behavior) [1]. According to large-scale national and international studies in Western countries, risky behaviors are prevalent among young people [1,2]. Particularly, risky behaviors are highest in late adolescence and early adulthood [3,4]. Moreover, youth engagement in risky behaviors poses significant public health problems, as they contribute to the leading causes of premature morbidity and mortality [5,6,7,8,9,10,11]. Accordingly, understanding the risk and protective factors for such behaviors is of substantial importance to researchers and policymakers. 

### 1.1. ACEs and Risky Behaviors Research in China

A large body of studies have confirmed that adverse childhood experiences (ACEs), which include child maltreatment and household dysfunctions, are significant predictors for various kinds of risky behaviors, such as involvement in violence [12,13], suicide ideation [14], sexual risky behaviors [15,16], substance use [17,18], dietary problems [19], and problematic media use [20,21,22].

Although current knowledge on ACEs and risky behaviors is still predominantly derived from Western countries, research interest in non-Western countries like China has grown in recent years. A few studies have revealed the impact of ACEs on risky behaviors among Chinese youth [23,24,25,26,27,28]. Most of these studies have confirmed a significant impact of ACEs on young people’s suicidal behaviors [23,24,25,26]. A smaller body of research has investigated the association between ACEs and Chinese college students’ drinking problems [27], as well as ACEs and violence victimization and perpetration among Chinese adolescents [28]. While informative, evidence is still limited on the relationships between ACEs and other types of risky behaviors among Chinese youth. Notably, the extant literature on this topic has rarely explored mediation and moderation mechanisms through which childhood adversities impact risky behaviors. 

### 1.2. The Mediating Effect of Perceived Stress

Stress is a consequence of ACEs as well as a risk factor for subsequent involvement in risky behaviors. Building upon a range of interdisciplinary human development science, researchers have suggested an eco-bio-developmental framework that illustrates how early adversity experiences can get under the skin and undermine an individual’s body stress response system [29]. The framework specifies that after exposure to ACEs, an individual’s stress coping and tolerance ability is dramatically disrupted, leading to high levels of stress reactivity, which is usually assessed as *perceived stress* [30]. Indeed, empirical studies from various populations have demonstrated that higher levels of ACEs are significantly associated with greater levels of perceived stress [30,31,32,33,34,35]. 

The eco-bio-developmental framework also posits how ACEs can lead to a range of health-threatening behaviors (e.g., substance abuse) through changes in stress response systems [29]. According to this framework, compared to those without exposure to ACEs, individuals with a history of ACEs may be more likely to engage in risky behaviors to attain temporary relief or to cope with toxic stress caused by ACEs. Results from prior research also support this hypothesis [36,37]. For example, a study with a nationally representative sample of current or former drinkers in the U.S. found that individuals with ACEs were more likely to drink to cope with stress than for pleasure or social reasons [36]. Similarly, Strine and colleagues [37] found that psychological distress, which occurs when stress is severe, prolonged, or both [38], mediates significant portions of alcohol problems among adults with ACEs exposure. In short, the extant body of research indicates that perceived stress operates as a mediating mechanism in the link between ACEs and risky behaviors. 

### 1.3. The Moderating Effect of Social Support

Although the associations between ACEs, perceived stress, and risky behaviors have been well-documented in prior research, it is also important to note that ACEs do not always result in prominent perceived stress and risky behaviors. Many researchers have hypothesized that protective factors, such as social support (i.e., the assistance provided to individuals who are coping with stressful events), has “stress-buffering” effects on adverse health outcomes [39,40]. Social support, particularly perceived social support (i.e., the perception that others provide support when there are stressful challenges), can lessen the effect of stress by increasing perceived coping skills and reducing the damaging impact of childhood adversities [39,41]. 

Despite the theoretical hypothesis, empirical studies have revealed complex and conflicting findings on the moderating effect of social support (See reviews by [42,43]). Some studies have confirmed the buffering role of social support against risky behaviors in the general population and ACEs populations [26,44,45,46]. For example, one study using data from the National Longitudinal Study of Adolescent to Adult Health revealed that social support weakened the association between exposure to childhood trauma and criminal justice involvement in adolescence [46]. Another study with a nationally representative sample of adolescents (aged 10–20) in China also found that social support buffered the association between ACEs and suicide attempts [26]. However, other empirical studies have indicated that the buffering effect of social support did not significantly reduce involvement in risky behaviors [47,48,49]. For instance, a recent study with 1421 youths aged 10 to 22 in Chicago neighborhoods suggested that youth-reported external social support did not buffer the effects of childhood exposure to family conflicts on substance use during adolescence [48]. Given the theoretical relevance of the role of social support in shielding individuals from the potentially harmful consequences of ACEs exposure and the inconsistent findings of the buffering impacts of social support in prior literature, further research that examines the buffering effect of social support is strongly warranted. 

### 1.4. Research Gaps in the Lliterature

Despite existing studies on ACEs and risky behaviors, significant research gaps exist in the literature. First, most of these ACEs studies only examined one or several types of risky behaviors. So far, no ACEs study has investigated a wide range of risky behaviors among the same group of people. However, researchers have confirmed that risky behaviors tend to co-occur instead of acting independently [50,51,52]. The study of ACEs and co-occurring multiple risky behaviors may help practitioners target a cluster of health-related behaviors caused by childhood trauma rather than addressing an individual risky behavior. Second, knowledge about ACEs and risky behaviors in China-the most populous country on earth is still limited. The scientific findings from Western countries may not be generalizable in China due to different social contexts [53]. Research on ACEs and risky behaviors grounded in China may help translate evidence into culturally appropriate prevention and intervention programs and services among Chinese people. Third, although many studies have confirmed the mediating role of perceived stress in the relation between ACEs and subsequent physical and mental health outcomes [30,54,55,56], there is minimal research that has examined the mediating effect of perceived stress in the link between ACEs and risky behaviors more broadly. Finally, notwithstanding the fact that theoretical hypothesis [39,40] assumes that social support has a buffering effect on the relations between ACEs, perceived stress, and adverse health outcomes, the moderating effect of social support still needs to be further examined in empirical research. Furthermore, no study has tested the mediation effect of perceived stress and moderation effect of social support on risky behaviors jointly. The moderated mediation analysis may help understand the complex ways through which perceived stress and social support influence risky behaviors. It may also provide implications for the intervening mechanisms of risky behaviors in practice [57]. 

### 1.5. Purposes of This Study

To fill in the above research gaps, this study aims to examine the associations between ACEs, perceived stress, and risky behaviors among a group of rural young adults in China and explores whether perceived social support diminishes those associations. Specifically, four research questions are addressed in the current study:

(1) What is the prevalence of risky behaviors among a group of Chinese rural young adults?
(2) Are ACEs significantly associated with Chinese rural young adults’ risky behaviors?
(3) Does perceived stress mediate the association between ACEs and risky behaviors?
(4) Does social support moderate the pathways from ACEs to risky behaviors through perceived stress?

## 2. Materials and Methods

### 2.1. Participants and Research Design

This study recruited 7986 rural high school graduates from six high schools in three different provinces of China (Hebei, Anhui, and Jiangsu) between 2016 and 2018. Three cohorts of high school graduates were recruited annually from each school using students’ private email addresses. In addition, a web-based survey was distributed to the participants via Qualtrics (Provo, UT, USA) when they turned 18 years old and graduated from high school.

The study survey asked about participants’ family background, childhood adversities, mental health problems, risky behaviors, and other health outcomes. Pre-and-post-survey reminders were sent to participants to promote their survey completion. Approximately 24% of the sample (n = 1888) could not be reached because the emails were undeliverable. Of the 6098 participants who could receive our survey emails, 1091 completed the survey. Thus, the net response rate is 18%.

Study participation was completely voluntary and confidential, and no personal identifying information was collected in this study. After completing the questionnaire, respondents received a 25 Yuan (about $3.80) Amazon China gift card. The study was approved by administrators of the six high schools and the institutional review board (IRB) at a public research university in the United States (please refer to Zhang et al. 2020 [14] to learn more details about the research design).

### 2.2. Measures

Adverse childhood experiences. Participants completed the Childhood Experiences Survey (CES, [58]), a measure of 10 conventional ACEs and seven other potential adversities that occurred before 18 years old. For more descriptions of the CES measure, please refer to Zhang et al. 2020 [14]. In this study, we used measures that resemble the 10 conventional ACEs [59], which include 5 types of child maltreatment (physical abuse, emotional abuse, sexual abuse, physical neglect, emotional neglect) and 5 types of household dysfunctions (parental mental illness, substance use, domestic violence, divorce/separation, incarceration).

Risky behaviors. In this study, seven types of risky behaviors among rural Chinese young adults were examined, including physical fighting, drinking, smoking, drug use, early sexual behaviors, digital media overuse, and suicide ideation. Measurement description of these risky behaviors is as follows. A total risky behavior score was also calculated with a range of 0–7.

Physical fighting. This was measured by one survey question: *During the past 12 months, how many times were you in a physical fight?* A response to “at least once” indicated physical fighting.

Drinking problem. Participants’ drinking problem was measured by the Alcohol Use Disorders Identification Test-Consumption (AUDIT-C), a 3-item alcohol screen that can reliably identify persons who are hazardous drinkers or have active alcohol use disorders [60]. The three items are: *How often did you have a drink containing alcohol in the past year; How many drinks containing alcohol did you have on a typical day when you were drinking in the past year; How often did you have six or more drinks on one occasion in the past year?* In the current sample, the internal consistency of AUDIT-C is 0.75. The AUDIT-C is scored on a scale of 0–12. In men, a score of 4 or more is considered positive for identifying hazardous drinking or active alcohol use disorders. In women, a score of 3 or more is considered positive [60]. AUDIT-C has also been validated in the Chinese samples [61,62].

Smoking problem. Two survey questions measured the smoking problem: *Have you smoked at least 100 cigarettes in your entire life? Do you now smoke cigarettes every day, some days, or not at all?* A confirmative response to the first question indicated a smoking problem. Answers “every day” or “some days” to the second question also showed a smoking problem. 

Drug use. Drug use was measured by one question: How many times in the past year have you used an illegal drug or used a prescription medication for non-medical reasons? The answer “at least once” indicated drug use. 

Early sexual behavior. This was measured by one survey question: *How old were you when you had consensual sexual intercourse for the first time?* According to empirical studies, most Chinese young people tended to have initial sexual behavior after 18 years old [63,64,65], thus, participants’ sexual first intercourse under 17 was coded as early sexual behavior in this study.

Digital Media overuse. Three questions measured digital media overuse: Over the past 30 days, how many hours per day did you play computer games or play video games? Over the past 30 days, how many hours per day did you spend on your smartphone or tablet like Ipad? Over the past 30 days, how many hours per day did you watch TV or movies? The range for digital media use per day is 0–15 h. Considering that there is still no consistent standard for defining overuse of digital media, we coded the hours of digital media use above 50th percentile (≥6 h) as overuse in this study.

Suicide ideation. The suicidal ideation was measured by a single question: *During the past 12 months, did you ever seriously consider attempting suicide?* An affirmative response to this question indicated suicide intention (1 = yes; 0 = no).

Global perceived stress. The 4-item version of the *Perceived Stress Scale* (PSS-4; [66]) was used as a global measure of perceived stress in the past month. The measure has been validated in Chinese-speaking samples [67]. In the current sample, the internal reliability of the PSS-4 was 0.74. A total score of perceived stress was calculated with a range of 0–16.

Perceived Social Support. Perceived social support was measured by the 4-item version of the *Medical Outcome Study Social Support Survey* (MOS-SSS), which provides an assessment of several domains of social support, including tangible support, emotional support, affective support, and positive support [68,69]. MOS-SSS was validated in Chinese studies as well [70,71]. In this study, the internal reliability of the abbreviated MOS-SSS is 0.77. A total score of MOS-SSS was also calculated with a range of 0–16.

Covariates. Demographic information reported by participants was used as covariates, including participant sex (1 = male); mother’s and father’s educational level (1 elementary school or less to 6 some college or more); mother’s and father’s employment status (1 = unemployed; 0 = employed full-time or part-time); the number of siblings (0, 1, 2, 3 or more), and if a parent had been a migrant worker before (1 = yes). In addition, an ordinal measure of family economic status was assessed by an economic ladder question [72,73], which asked participants to compare their family’s economic situation to that of other families on a scale from 1 (poorest) to 10 (richest). 

### 2.3. Data Analysis

Statistical analyses were performed using SPSS version 24 and involved four main procedures. First, a descriptive analysis was conducted to estimate the means and proportions of all study variables. Second, phi (ϕ) coefficients were produced from correlation analysis of ACEs, perceived stress, perceived social support, and total risky behaviors. Third, multivariate regression models were run to test associations between ACEs and different risky behaviors. All dichotomous outcomes were analyzed with logistic regression, and continuous measure was analyzed with Ordinary Least Squares (OLS) regression. All multivariate models controlled for sex, maternal and paternal education, maternal and paternal employment, family economic status, the number of siblings, and parent had been a migrant worker or not. Last, moderated mediation analysis was applied to examine the associations among ACEs, perceived stress, risky behaviors, and perceived social support. The moderated mediation analysis is a method that integrates moderation and mediation [74]. The analyses were performed using IBM SPSS software (version 24). The mediation model (Model 4) and the moderated mediation model (Model 58) were tested using the PROCESS macro [75]. Covariates were included in both models as control variables. Bootstrapping (n = 5000) and 95% confidence intervals were used to assess the indirect effects [76]. Importantly, using bootstrapping, no assumptions about the shape of the sampling distribution of the statistic are necessary when conducting inferential tests [77]. 

## 3. Results

Results from descriptive analyses are presented in Table 1. 25.0% reported zero ACEs, 29.1% reported 1 ACE, 21.5% endorsed 2 ACEs, 13.2% reported 3 ACEs, and 11.2% of the sample had four or more ACEs. Respondents reported the following prevalence of each risky behavior: 9.7% physical fighting, 7.4% drinking problem, 10.0% smoking problem, 0.9% drug use, 3.4% early sexual behavior, 58.7% social media overuse, 14.2% suicidal ideation. Furthermore, 29.5% of participants reported no risky behaviors at all, 46.9% one risky behavior, 16.4% two risky behaviors, and 7.2% three or more risky behaviors (not shown in table). The mean of total perceived stress is 6.1 (SD = 2.7), and the mean of total perceived social support is 11.2 (SD = 3.5). 

Table 2 shows the correlations between total ACEs score, perceived stress, perceived social support, and total risky behaviors. Results indicated that all these variables were significantly correlated with each other. The largest coefficient observed was the correlation between perceived stress and perceived social support (r = −0.35). 

Table 3 presents results from the multivariate regression models. Results showed that with increasing ACEs values, there was an increased likelihood of risky behaviors. For instance, the coefficient for the total risky behaviors score showed that the magnitude of the association between ACEs and risky behaviors generally increased alongside accumulating ACES (1 ACE: B *=* 0.20; 95% CI = [0.05, 0.36]; 2 ACEs: B *=* 0.34; 95% CI = [0.17, 0.51]; 3 ACEs: B *=* 0.47; 95% CI = [0.28, 0.67]; ≥4 ACEs: B *=* 0.85; 95% CI = [0.64, 1.06]. The same general pattern is found across each specific risky behavior except drug use, with 3 ACEs or ≥4 ACEs in particular consistently having the strongest associations in terms of magnitude and statistical significance to the specific risky behavior. For drug use, only 0.9% of participants (n = 9) reported drug use, so we tested the effect of total ACEs score on drug use, and found the effect was also statistically significant (B = 1.81, 95% CI = [1.16, 2.80]).

**Figure 1:**
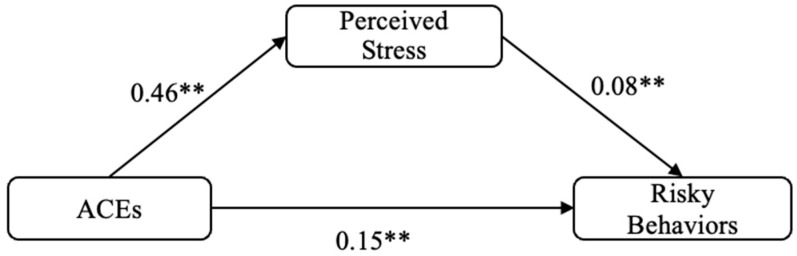
Mediation analysis model linking ACEs to risky behaviors through perceived stress. ** *p* < 0.01.

Figure 1 reveals the results of mediation analysis between ACEs total score and risky behaviors total score via perceived stress. The effects of ACEs on risky behaviors (b = 0.15, se = 0.02, t = 7.12, *p* < 0.001) and perceived stress (b = 0.46, se = 0.06, t = 8,12, *p* < 0.001) were both statistically significant. Perceived stress also had a significant association with risky behaviors (b = 0.08, se = 0.01, t = 6.84; *p* < 0.001). The total effect of ACEs on risky behaviors was significant (b = 0.18, se = 0.02, t = 8.97, *p* < 0.001), and the indirect effect of ACEs on risky behaviors via perceived stress was also significant (b = 0.04, se = 0.01, 95% CI = [0.02, 0.05]). Over 22% of ACEs effect on risky behaviors was mediated by perceived stress.

**Figure 2:**
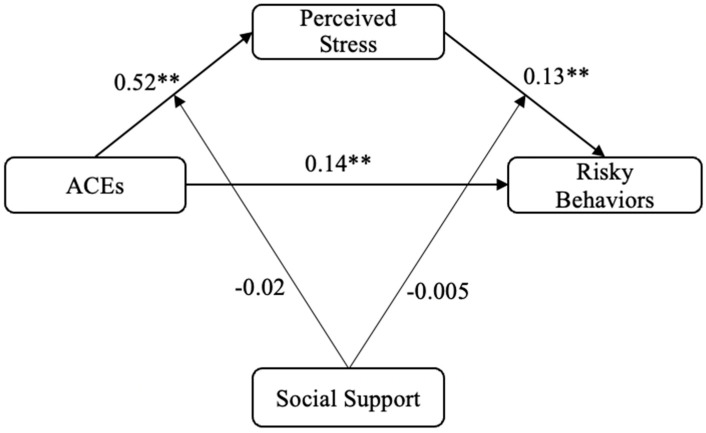
Moderated Mediation Analysis Model. ** *p* < 0.01.

Figure 2 shows the results of the moderated mediation model. The findings confirm that there was significant direct effect of ACEs on risky behaviors, and perceived stress also significantly mediated the relationship between ACEs and risky behaviors. However, no significant moderating effect of social support was found. Specifically, social support did not significantly moderate the effect of ACEs on perceived stress (β = −0.02, se = 0.01, t = −1.21, 95% CI = [−0.05, 0.01], *p* = 0.23), and the effect of perceived stress on risky behaviors either (β = −0.005, se = 0.003, t = −1.50, 95% CI = [−0.01, 0.001], *p* = 0.13).

## 4. Discussion

### 4.1. Discussion of Study Results

Both ACEs and risky behaviors are major public health issues that exhibit substantial influence on the wellbeing of young adults. For a long time, scholars have speculated that perceived stress may be an effective mechanism through which ACEs cascade their impact on risky behaviors. Social support has also been assumed to buffer the effect of perceived stress. However, to the best of our knowledge, no study had tested these hypotheses in one study. This study expanded upon extant research by assessing the influence of ACEs on different risky behaviors among a sample of rural young adults in China, as well as providing a novel assessment of the joint moderation-mediation effect of perceived stress and social support in this association. The study yielded several significant findings.

First, this study revealed that both ACEs and risky behaviors were very prevalent among rural Chinese young adults. For instance, 75% of participants had exposed to at least 1 ACE, and nearly 70% had developed at least 1 risky behavior. This finding corroborates other researchers’ conclusions that rural children in China tend to have more adversities and problems, as most of them have poorer family and school conditions compared to their urban counterparts [14,78]. In particular, many rural children have experienced long-term parental separation, because one or both parents migrated to cities to make a living [79,80]. Undoubtedly, the unfavorable development environment incurs multiple childhood adversities, which may lead to a variety of negative outcomes. 

Second, the results detailed that accumulating exposure to ACEs, particularly three or four or more ACEs, was robustly associated with elevated likelihood of engaging in risky behaviors. These results are consistent with prior research in China that has documented that ACEs are related to single type of risky behavior, including drinking, violent victimization and perpetration, and suicidal ideation [23,24,25,26,27,28]. However, the current study further expanded the existing research in two aspects: (1) It assessed the connection between ACEs and a variety of different risky behaviors among the same group of people, and demonstrated that vulnerable population was at risk for multiple risky behaviors rather than single type; (2) This study especially explored the relationships between ACEs and social media overuse and early sexual behaviors, two health-harming problems that were rarely examined in China ACEs studies. A few Western studies have found that childhood adversities were significantly associated with addition to social media [81] and early sex initiation [82,83,84,85]. Our study confirmed these findings in a sample of rural Chinese young people.

Third, drawing on an eco-bio-developmental framework [29], as well as research detailing how stress operates as a mediating pathway between ACEs and substance use [36,37], we analyzed the mediating role of perceived stress in the association between ACEs and total types of risky behaviors. Our study result showed that perceived stress mediated the link between ACEs and risky behaviors, with approximately 22% of the relationship between ACEs on risky behaviors operating indirectly through perceived stress. Based on our findings and other research evidence, it seems perceived stress plays a relatively stable role in mediating the relationship between ACEs and risky behaviors.

Fourth, based on the stress-buffering model of social support [39,40], we also assessed if young adults’ perceptions of social support moderated the association between ACEs and risky behaviors via perceived stress. Our moderated mediation analysis first confirmed again that perceived stress significantly mediated the association between ACEs and risky behaviors. However, respondents’ perceived social support did not significantly moderate the relationship between ACEs and perceived stress, or the relationship between perceived stress and risky behaviors, indicating that accumulating exposure to ACEs resulted in increased stress and involvement of risky behaviors, regardless of the degree of social support that an individual receives. This study finding is consistent with a small body of research which also provides evidence that social support has limited efficacy in buffering the harms [47,48,49]. However, other studies have confirmed the moderation effect of social support, especially in ACEs research [26,44,45,46]. The inconsistent findings may be attributable to different research methods adopted in studies, such as different samples, different measurements of social support, and different statistical techniques [42,43,47,48,49]. Replicated research is warranted to further test if social support has a stress-buffering effect or not.

### 4.2. Limitations

It is essential to highlight some limitations when interpreting the results. First, this study used a non-probability sample, which included high school graduates from rural areas of China, therefore, the findings may not be generalized to other urban contexts or younger students. Generalizability also may be limited by the study’s low response rate (18%), which is a common challenge with web-based surveys [86,87]. Second, the study relied on self-report data, which may be subject to recall bias or social desirability [88]. Third, due to the retrospective, cross-sectional design, no causal inferences should be made. It is plausible, for example, that participants’ perceived stress could affect their perception of childhood trauma, social support, and risky behaviors. 

### 4.3. Implications and Future Directions 

The results of the current study carry significant implications for directing future research on this topic. Notably, despite representing nearly one-fifth of the global population, research on the influence of ACEs on Chinese persons over their life-course remains understudied [14,89]. This is especially the case among rural Chinese persons, which compose approximately 40% of the country’s population. Therefore, more large-scale public health research among Chinese rural populations is needed. Considering the unclear moderating effect of social support in prior literature, future scholars should also continue to test the buffering hypothesis of social support using more consistent research methodologies and data. Moreover, this study only explored the mechanisms of perceived stress and social support through which ACEs might impact risky behaviors. Future research should examine other potential mechanisms such as depression, anxiety, and resilience that may also mediate or moderate the association between ACEs and risky behaviors. 

Besides the directions for future research, our study findings also provide implications for culturally appropriate practice, to improve the wellbeing of rural people in China. Our results showed that ACEs and risky behaviors were both prevalent among Chinese rural young people. One practice option may be using the Health and Family Planning Commission (HFPC) in China to deliver the home visiting programs that can provide parental education and prevention efforts to rural households with high risk of maltreatment and dysfunctions. Please see more discussions on this in Zhang et al. 2020 [14]. Such programs have shown the potential for reducing child maltreatment, improving development, and reducing risky behaviors later in life [90,91]. Furthermore, since most of our participants attended boarding high schools, school-based monitoring, educational workshops, counselling services and other potential interventions are also needed for those young people who have displayed significant risky behaviors [92,93].

The study findings also demonstrated that the association between ACEs and risky behaviors operates in part through perceived stress. This finding suggests that in cases where ACEs cannot be prevented, it may be helpful to design and implement interventions that can reduce stress as a means to mitigate the harms that stem from ACEs exposure. For example, an avenue is through the expansion of mental health treatment in rural China. Estimates suggest that sizeable portions of the Chinese population have a diagnosable mental health or psychiatric disorder, yet most individuals never seek treatment [94]. Accordingly, expanding efforts that can reform the mental health infrastructure in China to deliver a greater access to mental health services, could reduce the unfavorable consequences caused by ACEs and promote Chinese people’s mental health and overall wellbeing greatly.

Although our study did not find the stress-buffering effect of social support, social support may still moderate environmental vulnerabilities and confer resilience to stress (see reviews by [42,43]). Consequentially, any interventions that aim to increase family, school, and community support for disadvantaged children and families are encouraged [95,96]. Additionally, another beneficial approach may be through the expansion of resilience training programs at school that aim to promote traits including positive emotions, active problem solving, and coping skills, considering that prior research has shown that psychological resilience can offer protective benefits against the adverse mental health consequences of ACEs [97,98]. 

## 5. Conclusions

The results of this study aimed to better understand the connection between ACEs and risky behaviors among an understudied population of rural young adults in China. The findings revealed that accumulating exposure to ACEs earlier increases the likelihood of involvement in a variety of risky behaviors in young adulthood, and this association operates in partially through perceived stress. From a theoretical standpoint, this work provides support for an eco-bio-developmental framework. From a practical standpoint, this study highlights the ability of ACEs to carry lasting harm among rural Chinese youth and suggests the need to develop interventions which can help mitigate the negative impacts of ACEs and the stress that stems from such experiences.

## Figures and Tables

**Table 1:** Description of Study Measures (N = 1019).

Variable	% or Mean (SD)
**Demographics**	
Age (range 18–21)	18.6 (0.8)
Sex (male = 1)	53.0
Father education (range 1–6)	3.1 (1.5)
Mother education (range 1–6)	2.5 (1.5)
Father unemployed	12.0
Mother unemployed	26.9
Father or mother a migrant worker	71.3
Number of siblings (range 0–4)	1.0 (0.9)
Family economic status (range 1–10)	4.1 (1.4)
**Risky Behaviors**	
Physical fighting	9.7
Drinking problem	7.4
Smoking problem	10.0
Drug use	0.9
Early sexual behavior	3.4
Digital media overuse	58.7
Suicidal ideation	14.2
Total risky behaviors (range 0–7)	1.0 (1.0)
**Perceived Stress (range 0–16)**	6.1 (2.7)
**Perceived Social Support (range 0–16)**	11.2 (3.5)
**Conventional ACEs**	
0 ACE	25.0
1 ACE	29.1
2 ACEs	21.5
3 ACEs	13.2
4 or more ACEs	11.2
Cumulative score (range 0–10)	1.6 (1.5)

**Table 2:** Correlations between ACEs, social support, perceived stress, and total risky behaviors.

	1.	2.	3.	4.
ACEs Index	-			
2. Social support	−0.20 **	-		
3. Perceived stress	0.27 **	−0.35 **	-	
4. Risky behaviors	0.26 **	−0.20 **	0.26 **	-

** *p* < 0.01.

**Table 3:** Multivariate Analysis of Associations between ACEs and Risky Behaviors.

Outcome	Number of ACEs	B or OR (95% CI)
Physical fighting	0	(referent)
	1	1.62 (0.76–3.45)
	2	3.16 (1.52–6.58) **
	3	3.01 (1.33–6.79) **
	≥4	3.29 (1.41–7.70) **
Drinking problem	0	(referent)
	1	1.03 (0.44–2.41)
	2	2.22 (1.00–4.90) *
	3	3.18 (1.40–7.24) **
	≥4	3.67 (1.56–8.65) **
Smoking problem	0	(referent)
	1	0.98 (0.48–2.00)
	2	2.15 (1.10–4.23) *
	3	2.01 (0.93–4.38)
	≥4	2.60 (1.16–5.86) *
Drug use	ACEs total score	1.81 (1.16–2.80) **
Sexual behavior	0	(referent)
	1	1.43 (0.33–6.09)
	2	3.03 (0.78–11.74)
	3	4.54 (1.14–18.15) *
	≥4	9.88 (2.64–37.00) **
Digital medial overuse	0	(referent)
	1	1.39 (0.97–2.00)
	2	1.05 (0.71–1.54)
	3	1.63 (1.02–2.60) *
	≥4	1.98 (1.19–3.27) **
Suicidal ideation	0	(referent)
	1	3.74 (1.80–7.74) **
	2	4.25 (2.00–9.05) **
	3	5.97 (2.72–13.10) **
	≥4	15.46 (7.27–32.89) **
Total risky behaviors	0	(referent)
	1	0.20 (0.05–0.36) **
	2	0.34 (0.17–0.51) **
	3	0.47 (0.28–0.67) **
	≥4	0.85 (0.64–1.06) **

Note. B = unstandardized coefficient. OR = odds ratio. CI = confidence interval. * *p* < 0.05, ** *p* < 0.01.

## Data Availability

The data involved in the study can be obtained from the corresponding author at reasonable request.
